# Herpes Simplex Pneumonitis Presenting As Acute Respiratory Distress Syndrome and Septic Shock

**DOI:** 10.7759/cureus.75075

**Published:** 2024-12-04

**Authors:** Ramakanth Pata, Bhanu Kosuru, Joanna Kristeva

**Affiliations:** 1 Pulmonary and Critical Care Medicine, CentraCare Health System, Saint Cloud, USA; 2 Pulmonary and Critical Care Medicine, One Brooklyn Health, New York, USA; 3 Pulmonary and Critical Care Medicine, University of Cincinnati Medical Center, Cincinatti, USA; 4 Internal Medicine, University of Pittsburgh Medical Center (UPMC) East, Monroeville, USA; 5 Internal Medicine, One Brooklyn Health, New York, USA

**Keywords:** acute respiratory distress syndrome (ards), acute respiratory failure, disseminated herpes simplex virus infection, herpes simplex virus, herpetic tracheobronchitis, hsv pneumonia, immunocompromised patients, neutropenic sepsis, septic shock, septic shock (ss)

## Abstract

We present a case report of a 72-year-old female with a history of stage III rectal adenocarcinoma undergoing chemotherapy who developed neutropenic sepsis and acute respiratory failure. The patient was admitted to the intensive care unit (ICU) due to worsening respiratory status and was subsequently diagnosed with disseminated herpes simplex virus (HSV) infection including acute respiratory distress syndrome (ARDS). This case highlights the challenges in diagnosing and managing HSV infection in critically ill patients and emphasizes the importance of early recognition and appropriate treatment in improving patient outcomes. This case underscores the significance of considering viral etiologies, such as HSV, in patients with unexplained respiratory symptoms presenting as ARDS.

## Introduction

Disseminated herpes simplex virus (HSV) infection is a rare but potentially life-threatening condition, especially in immunocompromised individuals, and is associated with high mortality [1,2]. HSV remains dormant in neural ganglion cells and is reactivated during immunosuppression. Typically, it causes upper respiratory tract infection but occasionally can lead to severe illness needing intensive care unit (ICU) admission. HSV may be isolated in respiratory specimens of many critically ill patients especially those who have been on mechanical ventilation greater than five days. It is difficult to differentiate whether the detection of HSV represents asymptomatic shedding or a true infection responsible for pneumonia [2]. HSV pneumonitis should be suspected in patients with acute respiratory distress syndrome (ARDS) with negative bronchoalveolar bacterial cultures and a negative respiratory viral panel. Here, we present a case of HSV pneumonitis presenting as ARDS and septic shock with a history of transient neutropenia secondary to chemotherapy. Bronchoalveolar lavage (BAL) and blood were positive for HSV PCR. Acyclovir was started with a good clinical response. 

## Case presentation

An elderly female patient, aged 72, was admitted to ICU for management of acute hypoxic respiratory failure. Her medical history included hypertension, gastro-esophageal reflux disease, chronic kidney disease stage IIIa (eGFR of 50 mL/min and baseline creatinine of 1.5 mg/dL), and a former tobacco use disorder (cigarette smoking, 30 pack years, quit eight years ago). Additionally, she had recently (three months ago) been diagnosed with stage III rectal adenocarcinoma and was undergoing chemotherapy with the FOLFOX (Folinic acid, Fluorouracil, Oxaliplatin) regimen.

Four days after completing the sixth cycle of chemotherapy, the patient presented to a suburban facility with symptoms of fever, malaise, loss of appetite, abdominal pain, and diarrhea and was subsequently admitted for neutropenic sepsis. Broad-spectrum antibiotics were immediately initiated; however, blood and urine cultures yielded negative results. Due to the progressive worsening of her respiratory status, she required intubation and was subsequently transferred to our facility for further care. Upon arrival, the patient's vital signs revealed a temperature of 101°F, a pulse of 110, a blood pressure of 80/60, and a saturation of 91% with 100% FiO_2_. The physical examination was notable for diffuse crepitations in bilateral lung fields. The initial chest radiographs were consistent with bilateral diffuse consolidation consistent with ARDS. Immediate resuscitation measures were taken, including the placement of a central line and an arterial line. Intravenous fluid resuscitation was done with normal saline at 20 mL/kg. Vasopressors including nor-epinephrine and vasopressin at 0.03 units/hr were initiated. Nor-epinephrine infusion was titrated to a mean arterial pressure (MAP) goal greater than 65 mmHG, requiring 0.3 to 0.4 mcg/kg/min. Initial ABG revealed a pH of 7.19 and PaCO_2_ of 38. The bedside echocardiogram indicated a grossly normal ejection fraction of 60-65% and an absence of preload responsiveness by the absence of a significant increase of velocity-time integral with passive leg raise. See Table 1 for laboratory workup. 

**Table 1:** Laboratory workup on the day of admission except for bronchoscopy specimens.

Parameter	Result	Reference range
Sodium	135 (L)	136-146 mmol/L
Potassium	4.5	3.5-5.1 mmol/L
Chloride	104	98-107 mmol/L
Total CO_2_ (HCO_3_)	12	22-29 mmol/L
Creatinine	2.31 (H)	0.57-1.11 mg/dL
Blood urea nitrogen	78.8 (H)	10.0-20.0 mg/dL
Calcium	8.7	8.6-10.5 mg/dL
Glucose	171 (H)	70-100 mg/dL
eGFR	22 (L)	≥60 mL/min/1.73m^2^
Hemoglobin	8.1 (L)	11.2-15.8 g/dL
WBC count	16.3 (H)	3.7-12.1×10^3^/uL
Platelets	37 (LL)	179-450×10^3^/uL
Magnesium	2.2	1.8-2.6 mg/dL
Phosphorus	4.5	2.9-5.2 mg/dL
INR	1.3 (H)	0.9-1.1
LDH	411 (H)	125-220 U/L
Blood culture	No growth	Negative
Nares	No MRSA	Negative
BAL - culture	Negative	Negative
BAL - HSV 1, PCR	Positive (A)	Negative
BAL - HSV 2, PCR	Negative	Negative
BAL - Legionella PCR	Negative	Not applicable
BAL - Aspergillus Ag	<0.500	<0.5 index
BAL - Cytomegalovirus PCR	Negative	Negative
HSV 1 PCR, blood	Positive (A)	Negative
HSV 2 PCR, blood	Negative	Negative
Peripheral blood smear	Value	Reference range
Coarse basophilic stippling	1+ (A)	None
Dacrocytes	1+ (A)	None
Platelet estimate	Decreased (A)	Adequate
Polychromasia	Present (A)	None

**Figure 1:**
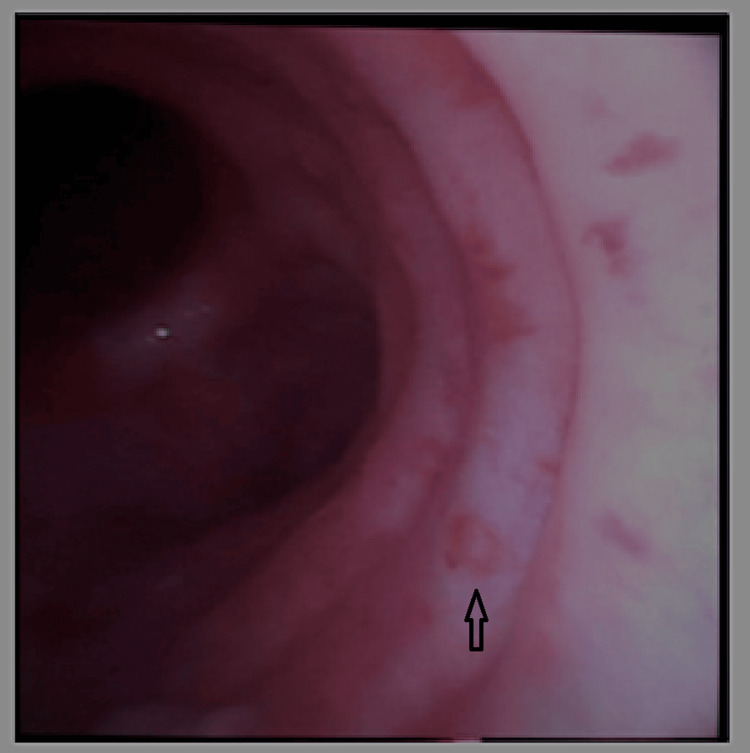
Bedside bronchoscopy revealed multiple erosions (arrow) with erythematous bases suspicious of herpetic tracheobronchitis.

The patient was found to be profoundly acidotic and exhibited acute kidney injury, leading to the immediate initiation of continuous renal replacement therapy (CRRT). Mechanical ventilation was managed using low tidal volume ventilation at 6 mL/kg predicted body weight (PBW) and positive end-expiratory pressure (PEEP) titration based on driving pressure. With a PEEP of 12 cmH_2_O, the FiO_2_ could be reduced to 60% with a PF ratio of 170, thus making prone positioning unnecessary. As the initial respiratory viral panel was negative, a bronchoscopy was done to obtain bronchoalveolar samples. A subsequent bedside bronchoscopy revealed multiple 2-5 mm erosions with erythematous bases diffusely in the tracheobronchial tree indicative of herpetic tracheobronchitis (see Figure 1).

**Figure 2:**
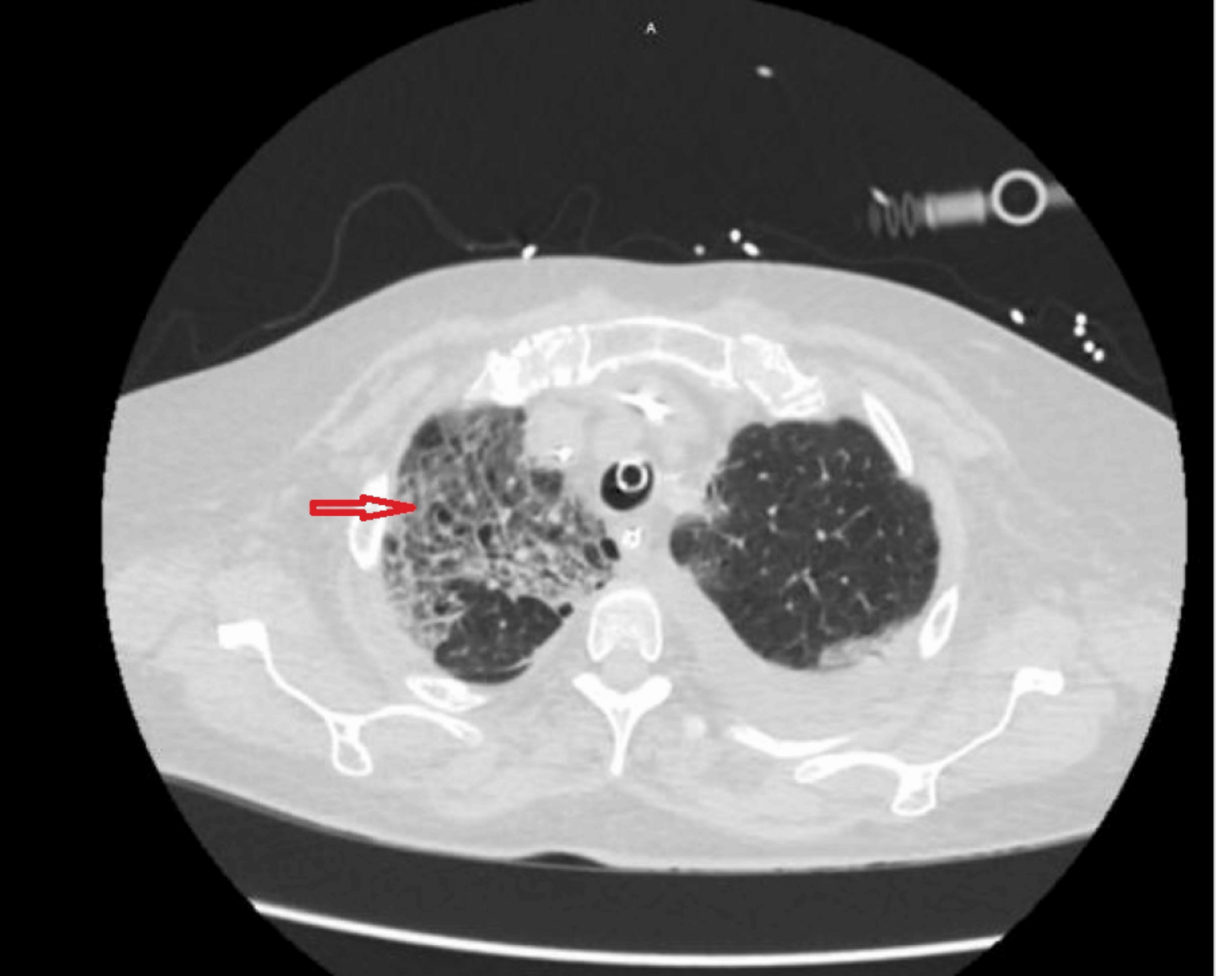
A chest CT scan (axial view, lung window) at the level of the trachea revealed patchy ground-glass opacities with interlobular septal thickening (arrow), primarily in the upper lobes. Centrilobular emphysema was also observed, predominantly in the upper lobes.

**Figure 3:**
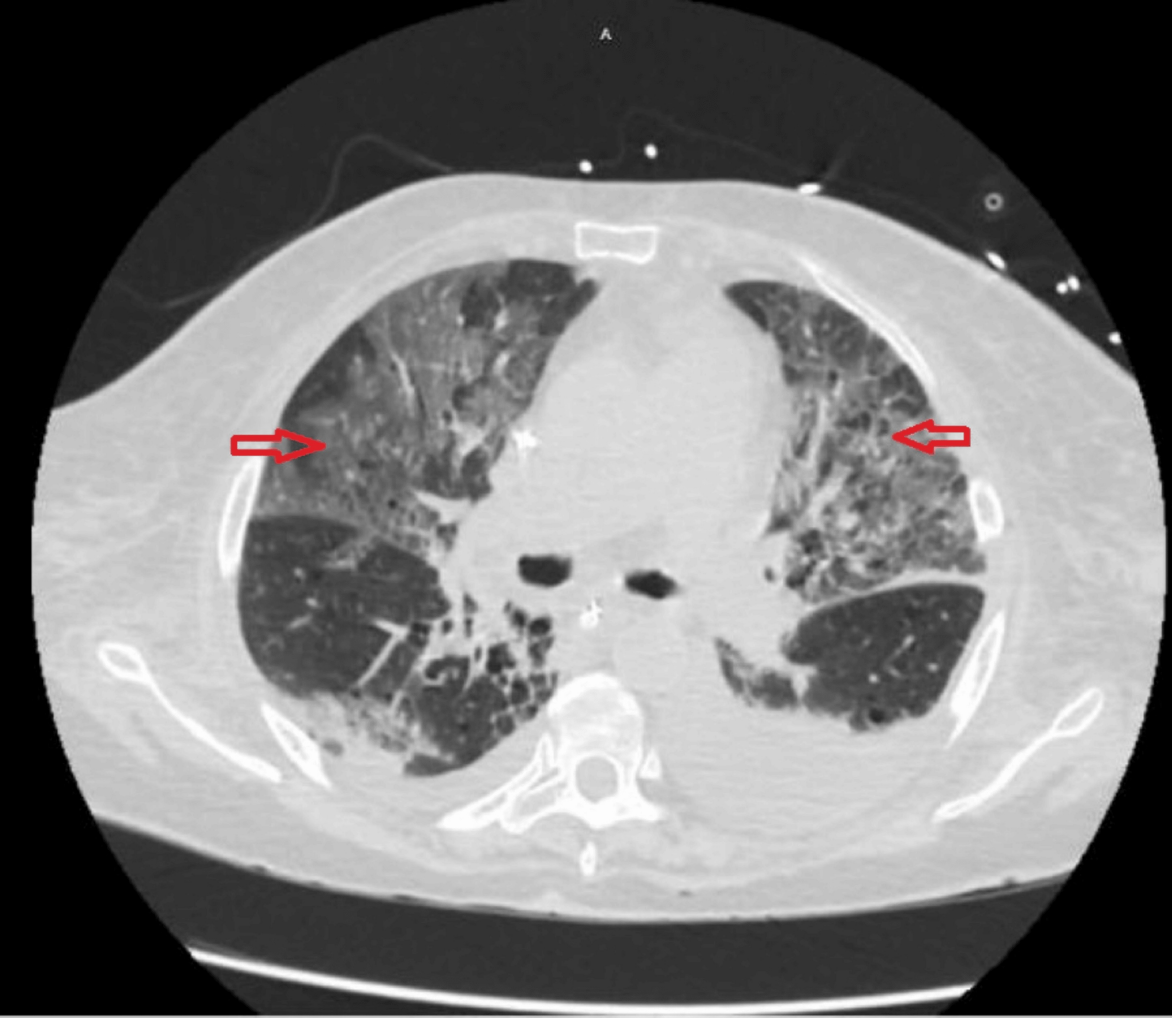
A chest CT scan (axial view, lung window) at the level of the pulmonary artery bifurcation revealed diffuse bilateral patchy ground-glass opacities with interlobular septal thickening, raising suspicion for pneumonitis. The ground-glass opacities (arrow) may indicate inflammation or alveolar hemorrhage. Bilateral pleural effusions with dependent consolidation were also noted, likely due to fluid overload secondary to acute kidney injury.

**Figure 4:**
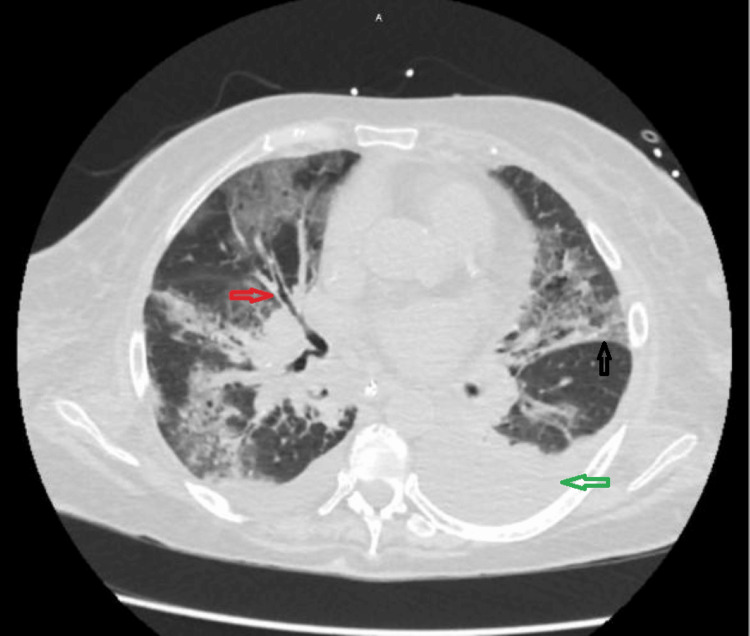
A chest CT scan (axial view, lung window) at the level of the left atrium demonstrated diffuse bilateral patchy ground-glass opacities with interlobular septal thickening, predominantly in the peribronchiovascular areas, raising suspicion for viral pneumonia. These findings suggest that the origin is primarily the airways with secondary viremia rather than the reverse. The ground-glass opacities (black arrow) are patchy and predominantly located in non-dependent areas, ruling out pulmonary edema as the cause. Evidence of traction bronchiectasis (red arrow) is also present, suggesting pulmonary fibrosis. Bilateral pleural effusions with dependent consolidation (green arrow) are noted, likely due to fluid overload secondary to acute kidney injury.

A CT chest scan was then performed to determine the extent of consolidation, which revealed diffuse bilateral consolidations and an interstitial pattern (see Figure 2). There was evidence of bilateral pleural effusions, consolidation (see Figure 3), and traction bronchiectasis (see Figure 4). Mild motion artifacts can be appreciated on all images. However, the respiratory viral panel yielded negative results.

Ventilator settings were extremely high, precluding a lung biopsy, which was, therefore, not performed. Empirical acyclovir therapy was initiated at a dose of 7.5 mg/kg (after adjusting for CRRT with eGFR of 22 mL/min) due to a high clinical suspicion of herpes pneumonitis.

BAL cytology revealed no tumor cells, with a cell differential of 95% neutrophils, 5% lymphocytes, 0% macrophages, and 0% eosinophils. Additionally, a monolayer preparation and cell block preparation revealed scattered benign and degenerative-appearing squamous and bronchial epithelial cells amid an abundance of acute inflammatory cells. Some of these cells exhibited glassy nuclear inclusions, occasional red granular inclusions, and focal multi-nucleation. The affected cells appeared to be squamous epithelial cells, suggesting a viral cytotoxic effect. While these features were suspicious for a possible HSV infection, other viral causes, including cytomegalovirus (CMV), could not be entirely excluded based on cytologic features alone. Immunohistochemical testing for CMV using an analyte-specific reagent antibody (a cocktail of two mouse monoclonal antibodies, DDG9 and CCH2, diluted 1-200) and heat-induced epitope retrieval (bond ER solution 2 for 20 minutes) with a polymer detection system yielded negative results, with an appropriate control (Leica Microsystems Bond Instrument). Further investigations for immunosuppression, including HIV and immunoglobulin deficiency, were negative, except for a brief episode of neutropenia on the day of presentation, which responded well to filgrastim administered at the outside facility. HIV testing was negative. Subsequently, HSV-1 PCR testing returned positive in both BAL and blood samples, with results available on day 2 of ICU admission. A CT scan of the head and fundoscopy revealed no lesions suggestive of herpes infection.

Infectious diseases were consulted, and it was recommended to continue the same antiviral therapy for disseminated herpes infection. The patient remained on vasopressors (norepinephrine and vasopressin) for four days to target a MAP of 70 mm of Hg. The course was further complicated by new-onset atrial fibrillation on day 4 of admission, necessitating amiodarone infusion for three days. Beta-blockers and calcium channel blockers were not administered due to the patient's septic shock. Anticoagulation was deemed inappropriate due to diffuse oozing in the tracheobronchial tree. CRRT was successfully terminated on day 4, with the patient experiencing good renal recovery as defined by urine output >500 mL/day. Mechanical ventilation was continued for a total of seven days, after which the patient was successfully extubated and transitioned to a high-flow nasal cannula (30 L/min of flow and 40% FiO_2_) following a two-hour spontaneous breathing trial. Acyclovir therapy was administered for a total of 14 days, and subsequent HSV PCR testing in the blood sample yielded negative results. The patient was also found to have critical illness neuromyopathy. Ultimately, the patient was discharged to a short-term rehab facility on the 12th day after admission. At the time of discharge, she required 2 L/min of supplemental oxygen. Further discussion with the patient revealed a history of episodes of herpetic labialis (cold sores) in the past, with the most recent occurrence being one week prior to admission. The patient was followed up in the post-ICU clinic for one month and is clinically doing well. 

## Discussion

This case highlights the challenges in diagnosing and managing disseminated (blood-stream infection) HSV infection in immunocompromised patients. The initial presentation with neutropenic sepsis and acute respiratory failure, coupled with negative blood and urine cultures, posed a diagnostic dilemma. However, bronchoscopy and subsequent testing confirmed herpetic tracheobronchitis and pneumonitis. The prompt initiation of antiviral therapy, along with supportive care, resulted in a favorable outcome. HSV infection most commonly involves the upper respiratory tract, causing herpes labialis (cold sores) or gingivostomatitis and pharyngitis. This is usually due to reactivation during periods of stress, trauma, and fever. Until then, it remains dormant in neural ganglion cells. However, in immunosuppressed individuals (especially cell-mediated immunity), reactivation may result in life-threatening infections [1]. For this reason, most transplant recipients frequently receive prophylaxis with acyclovir. Our patient received chemotherapy for cancer-causing transient neutropenia, which must have caused the reactivation of the dormant herpes virus. The pathogenesis of HSV pneumonia is thought to occur from aspiration of salivary secretions and, during the transit, can result in pharyngo-tracheobronchitis [2]. On further questioning, our patient had a history of herpes labialis occurring one week prior to the hospital admission. Bronchoscopy performed during the ICU admission clearly demonstrated multiple 2-5 mm erosions suggestive of herpetic tracheobronchitis. Herpes simplex pneumonia presents with prodromal symptoms such as fever, myalgias, and GI symptoms. Our patient presented initially with complaints of abdominal pain and diarrhea and, with evidence of neutropenia, was empirically initially treated for neutropenic enterocolitis and received broad-spectrum antibiotics. This was followed by progressive respiratory failure, likely representing herpes pneumonitis. Blood cultures and CT abdomen pelvis were negative. Disseminated herpetic infection should be strongly in patients with neutropenia who do not respond to broad-spectrum antibiotics. In our case, HSV pneumonitis resulted in severe ARDS needing mechanical ventilation.

HSV is also frequently isolated in respiratory specimens of critically ill patients (5-64%), especially with prolonged intubation of >5 days [3,4]. In many circumstances, this represents a carrier state (referred to as innocent bystander due to asymptomatic shedding) rather than pneumonia. If HSV is the cause of pneumonia rather than being a carrier state, the prognosis is guarded [5]. Unwarranted therapy carries the risk of organ failure due to the drug's narrow therapeutic index, more so in critically ill patients who are on organ support [3]. Therefore, whether to treat a positive HSV sample or not is challenging, especially in critically ill patients. Of note, the traditional respiratory viral panel does not include HSV, and hence, a negative nasopharyngeal swab should not deter from suspecting herpes pneumonia. Serology is rarely useful as it cannot distinguish past from current infection. A bronchoscope is recommended not only to obtain bronchial washings or BAL, which represent true lower respiratory samples, but also the presence of tracheobronchitis like in our case supports early suspicion and almost always warrants acyclovir therapy [4,5]. HSV PCR should be obtained on blood samples in all cases of HSV pneumonia, as frequently disseminated HSV infection either as a cause or a consequence can be seen. For the same reason, imaging of the head and fundoscopy can establish the degree of dissemination. This is important as the duration of therapy may be different [6,7]. Our patient also had a positive HSV PCR on a blood sample representing disseminated herpes infection. Although viral cultures were traditionally considered as gold standard, PCR has become the test of choice due to its 100% sensitivity [6]. Like in our case, cytopathological changes seen on BAL almost always warrant therapy. Lung biopsy, if positive can confirm HSV pneumonia, but a negative result does not exclude. In critically ill patients, lung biopsy can be hazardous due to high ventilator settings [8].

Acyclovir therapy should be considered in all cases of positive blood samples or positive respiratory samples along with cytopathological changes on BAL or high viral load or if there is no other cause identified as the cause of respiratory failure [4,5,9]. In our case, cytopathological changes were not only seen on the BAL sample, but HSV was positive on the blood sample. Without therapy, the mortality rates can be as high as 30-60% [10].

There is no evidence to suggest the recommended dose and duration of acyclovir specifically for HSV pneumonitis. Most intensivists employ a dose recommended for HSV encephalitis of 10 mg/kg IV every eight hours. However, the drug is really cleared due to its low protein binding capacity and high water solubility. Hence, the dose must be adjusted if the patient is on renal replacement therapy. We have employed a dose of 7.5 mg/kg in our patient as she was on CRRT [11]. Steroids should be considered in severe ARDS to prevent fibrosis or to treat complications such as organizing pneumonia [12,13,14]. However, steroid administration in mild pneumonia can result in fatal hepatitis and dissemination. We have not considered steroid therapy for the risk of dissemination.

Older age, smoking history, and prolonged intubation are associated with worse outcomes, even in immune-competent critically ill patients [15]. Rather, some studies have shown that a positive HSV sample is a marker of severity regardless of immunocompetence [5]. Mortality of HSV pneumonia is reported at around 60% [5,16,17]. Our case has a positive outcome, likely due to earlier initiation of antiviral therapy due to the presence of herpetic erosions on the tracheobronchial tree.

The case described above had multiple circular erosions with an erythematous base in the tracheobronchial tree on bronchoscopy suggestive of tracheobronchitis. In addition, she was intubated for respiratory failure resulting from ARDS caused by HSV. A history of herpes labialis and herpetic tracheobronchitis a week prior, along with CT chest evidence of viral pneumonitis, is consistent with aspiration of salivary secretions as the cause of respiratory failure. Transient neutropenia would have been the risk factor or dissemination including the isolation of HSV-1 in the blood.

## Conclusions

In patients with neutropenia, even if transient, HSV pneumonitis should be considered in those presenting with ARDS and septic shock, especially if other diagnostic workups remain negative. It is important to note that a respiratory viral panel does not routinely include HSV testing; therefore, a virus-specific PCR test must be requested. Multiple circular erosions on bronchoscopy are almost always indicative of either HSV or CMV, and early initiation of antiviral therapy can reduce mortality.
